# Distalization of the mandibular first molar with clear aligners: a 3D finite element study

**DOI:** 10.3389/fbioe.2025.1665588

**Published:** 2025-11-13

**Authors:** Fujia Kang, Yifei Xu, Xinyan Zhao, Zhen Lu, Dongning Li, Jiamin Zhao, Yibao Li, Menghong Li, Kun Qi

**Affiliations:** 1 Key Laboratory of Shaanxi Province for Craniofacial Precision Medicine Research, College of Stomatology, Xi’an Jiaotong University, Xi’an, Shaanxi, China; 2 Department of Orthodontics, College of Stomatology, Xi’an Jiaotong University, Xi’an, Shaanxi, China; 3 Department of Oral Anatomy and Physiology and TMD, School of Stomatology, Air Force Medical University, Xi’an, Shaanxi, China; 4 School of Mathematics and Statistics, Xi’an Jiaotong University, Xi’an, Shaanxi, China

**Keywords:** clear aligner, tooth movement, molar distalization, finite element analysis, orthodontics

## Abstract

**Introduction:**

This study aims to analyze the impact of varying initial positions of the mandibular first molar and attachment configurations on tooth movement by finite element analysis (FEA).

**Methods:**

A three-dimensional (3D) model was constructed, including the mandible, dentition, periodontal ligaments (PDLs), clear aligners (CAs), and attachments. The second molar was initially positioned 3.2 mm distally. Groups were defined as follows: no attachment, attachment on the second molar (second molar ATT), and attachment on the first molar (first molar ATT). In each group, the first molar’s initial position relative to the second premolar varied (0 mm, 1 mm, 2 mm, and 3 mm). The displacement was extracted for result analysis.

**Results:**

With distal movement of the first molar’s initial position, distal displacement of the first molar, mesial displacement of the premolars, and labial displacement of the anterior teeth increased from 9.18e^−2^ to 1.12e^−1^ mm, from 1.32e^−2^ to 4.16e^−2^ mm, and from 4.07e^−2^ to 4.69e^−2^ mm, respectively, while mesial displacement of the second molar decreased from 4.59e^−2^ to 2.96e^−2^ mm in the NO ATT group. Displacement of the first molar, premolars, and anterior teeth in the first molar ATT group was greater than that in the other groups. For the second molar, the most significant mesial displacement was observed in the second molar ATT group.

**Conclusion:**

Without additional anchorage, the distal displacement of the first molar is concomitant with the opposite movement of other teeth in the dentition. With distal movement of the first molar’s initial position, the movement efficiency of the mandibular first molar increased. Therefore, increased step distance in precedence can be designed to enhance the overall movement efficiency.

## Introduction

Mandibular molar distalization serves as a method to increase the lower arch length, thereby providing space in non-extraction treatments. In particular, for patients with mild to moderate Angle Class III malocclusion and mild crowding of the mandibular dentition without severe skeletal deformities, mandibular molar distalization is usually selected to adjust the occlusion relationship and achieve an esthetic facial profile (Park et al., 2024; Inchingolo et al., 2023; Martina et al., 2024).

There are some traditional methods available for mandibular molar distalization, but their use is often limited by patient discomfort, poor compliance (Nevant et al., 1991; Griswold et al., 2022), and tipping movement of molars (Wang et al., 2024). For example, the lip bumper relies on cooperation from patients and inevitably causes lip discomfort (Nevant et al., 1991; Griswold et al., 2022). Moreover, the lower-lingual arch appliance impedes tongue mobility and results in opposite movement of anterior teeth (Ciftci et al., 2018; Arevalo et al., 2020). Furthermore, the headgear distalizes the molar using the external traction device anchored around the neck, which is clumsy (Osvaldik-Trapl and Droschl, 1978; Altug et al., 2005). As a fixed appliance, the multi-loop edgewise arch wire, which is time-consuming for bending, can achieve only crown distalization rather than bodily movement of the molar (Wang et al., 2024; Baek et al., 2008; He et al., 2013). Considering the limitations mentioned above, novel and practical methods for molar distalization are required. With the progress of computer-aided design and manufacturing, clear aligners (CAs) were introduced and became popular for their esthetics and comfort (Weir, 2017). More importantly, the material properties of deformation and elastic rebound are key mechanical advantages that CAs leverage for molar distalization.

At present, the high efficiency of upper molar distalization by CAs has been confirmed by many studies (Robertson et al., 2020), and bodily distalization of up to 1.5 mm has been reported to have the highest predictability (88%) (Ke et al., 2019). However, the research on the distalization of mandibular molars remains scarce. The effectiveness of the entire molar distalization stage is highly dependent on the geometry of the CAs, which changes at each treatment step and directly influences distalization efficiency. Specifically, when the first molar moves distally, the CAs mesial to the first molar become longer, and those distal to the first molar become shorter. Except for the shape of the CAs, the efficiency of molar distalization is also influenced by the application of attachments. For instance, the vertical rectangular attachment can reduce mesiodistal tipping tendency during maxillary molar distalization by increasing the contact area between the CAs and the teeth (Ayidaga and Kamiloglu, 2021). The gradual worsening of attachment wear compromised the efficiency of tooth movement (Li and Yang, 2024). Given that the width of a molar crown is typically greater than its height, a horizontal rectangular attachment theoretically provides a larger contact area than a vertical attachment. In this study, the horizontal rectangular attachment was used, and the efficiency of molar distalization with different configurations was compared.

Therefore, we hypothesize that both initial positions and attachment configurations influence the mandibular molar distalization when the second molar is repositioned to its target position. The maximum and average displacement of the crown and root of all teeth in the mandibular were extracted and compared. The findings of this study will provide insights for high-efficiency mandibular molar distalization.

## Materials and methods

### FEA model construction

**FIGURE 1:**
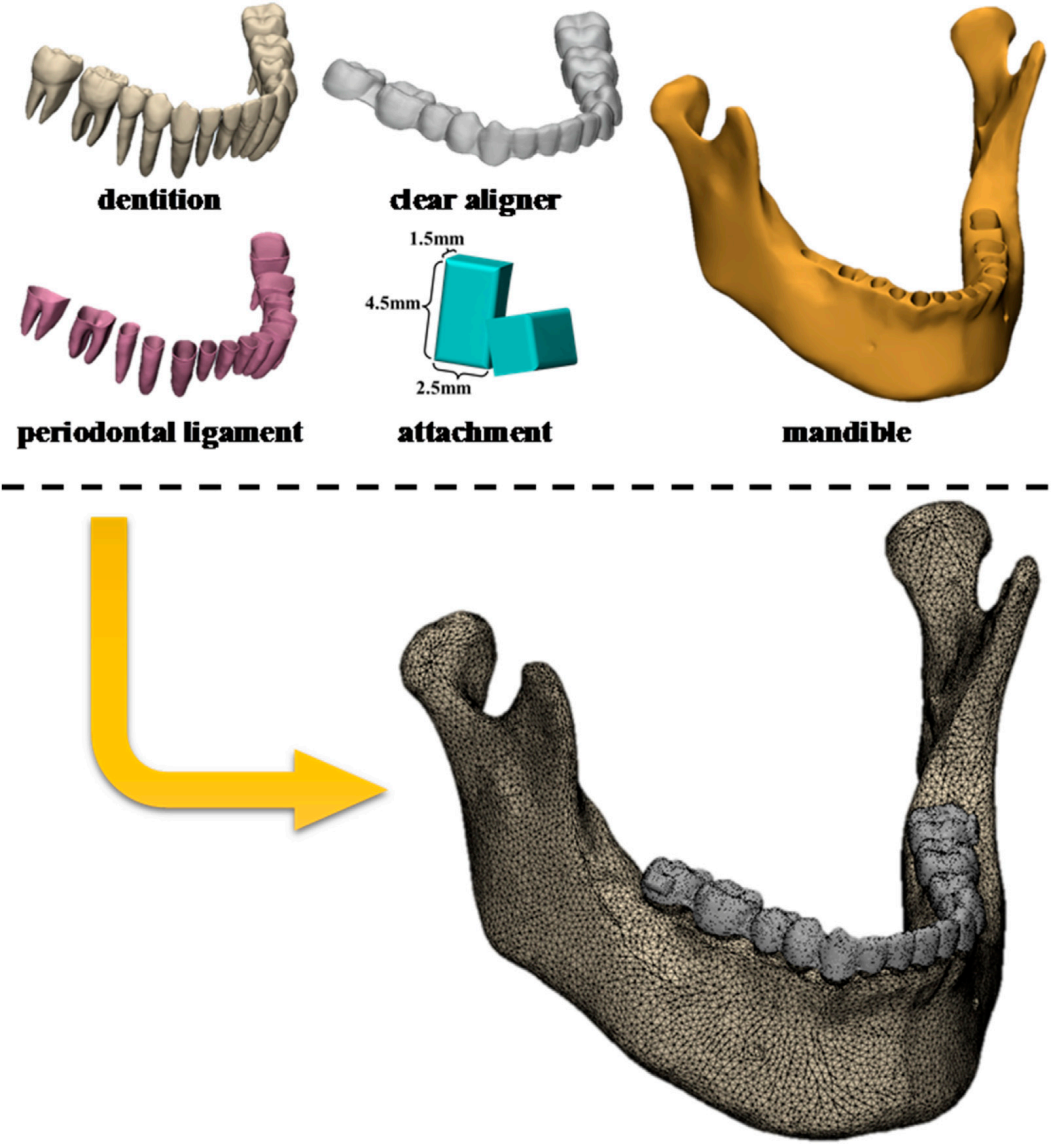
Process of finite element modeling.

This study has been approved by the Ethics Committee of the Stomatological Hospital of Xi’an Jiaotong University (Approval No. KY-OT-20240063). A CBCT scan (Planmeca ProMax 3D, Finland) was obtained from a patient with mild Angle’s Class III malocclusion who was treated with mandibular molar distalization. The data were exported as digital imaging and communications in medicine (DICOM) files for modeling. The appropriate grayscale thresholds were set in Mimics Research software (v21.0, Materialise, Belgium) to segment the image and generate the external surfaces of the mandible and the dentition model. The 3-Matic Research software program (v13.0, Materialise, Belgium) was used for PDL, CA, and attachment modeling. The PDLs were modeled by offsetting the external surface of the roots with an average thickness of 0.2 mm. Similarly, the CAs were modeled by expanding the outer surface of the target dentition with an average thickness of 0.7 mm. The horizontal rectangular attachment (4.5 mm in width, 2.5 mm in height, and 1.5 mm in thickness) was designed and positioned on the buccal surface of the crown, as shown in Figure 1.

### Grouping and material property assigning

The mandibular second molar was positioned 3.2 mm distal from the first molar, aligning with the occlusal plane defined by the buccal cusps of the mandibular molars. The models were categorized into three groups based on attachment configurations.

- No attachment (NO ATT).
- The attachment positioned on the mandibular second molar crown (second molar ATT).
- The attachment positioned on the mandibular first molar crown (first molar ATT).

**FIGURE 2:**
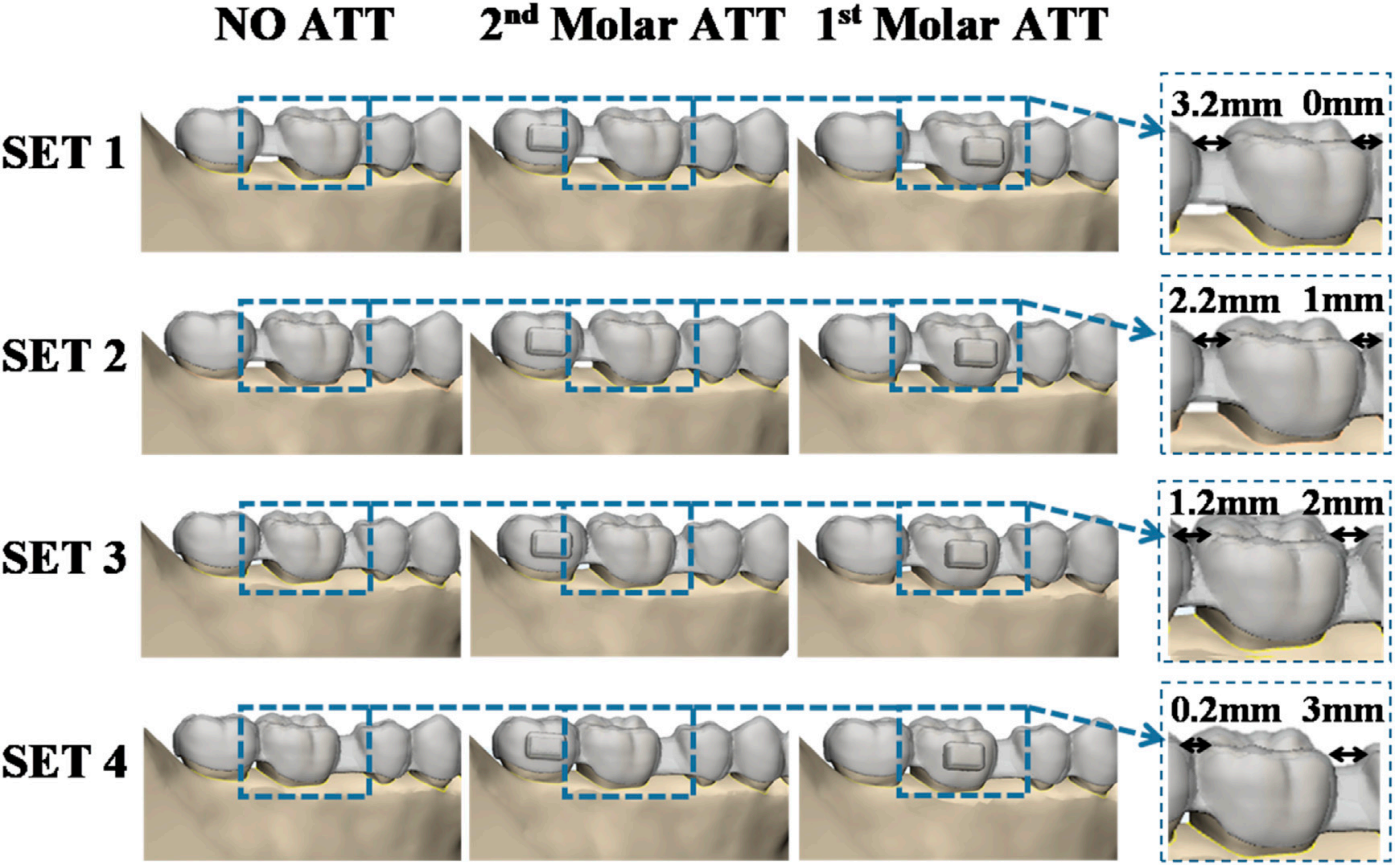
Diagram of various settings.

For each group, the first molar was initially positioned at distances of 0 mm, 1 mm, 2 mm, and 3 mm from the second premolar, which defined the subsets SET 1, SET 2, SET 3, and SET 4, respectively. In addition, the step distance was set at 0.2 mm (as shown in Figure 2).

The meshing of all the components was conducted in 3-Matic Research software with 0.2 mm edge length for PDLs, 2 mm for teeth, CAs, and mandible. The nodes between the teeth, PDLs, and mandible were set to be shared. The process produced a total of 1,425,619 nodes and 1,164,810 elements. The resulting files were imported into ANSYS Workbench 19.2 (ANSYS, United States). All structures involved in this study were considered to be linear, elastic, isotropic, and homogeneous materials. Given that the biomechanical behavior of the alveolar bone and teeth under physiological loading approximates linear elasticity, the use of a linear elastic model is a validated approach for finite element analysis in orthodontic applications. Material parameters were derived from previous literature (Cortona et al., 2020; Hong et al., 2021; Seo et al., 2021; Liu et al., 2021; Jia et al., 2023; Ma and Li, 2021; Kang et al., 2023), as referenced in Table 1.

**TABLE 1:** Material properties of the FEA model.

Biomaterial	Young’s modulus (MPa)	Poisson’s ratio
Tooth	19,600	0.3
Alveolar bone	13,700	0.3
PDL	0.68	0.45
CA	528	0.36

### Boundary constraints and contact conditions

A bonding connection was established between the surfaces of roots, PDLs, and mandible and between the attachments and the crowns. A frictional condition was constructed between the crowns and CAs. The friction coefficient was set at 0.2 (20). The lower surface and condyle of the mandible were set as fixed to restrict all degrees of freedom. The “two-step method” was adopted. Specifically, the mandibular first molars were moved from the target positions back to the initial positions, and then the stress of CAs was calculated and applied to the pre-orthodontic dentition as the orthodontic force.

### Coordinate system setting

**FIGURE 3:**
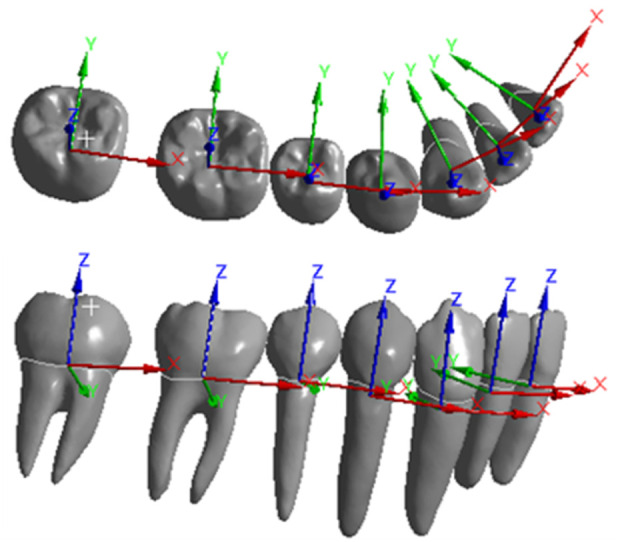
Local coordinate system for each tooth.

As the movement patterns were similar for the corresponding teeth on the left and right sides, the local coordinate system for the right mandibular teeth was established to define the x, y, and z axes (as represented in Figure 3). The origin of the coordinate system was located at the center of the tooth. The z-axis was defined along the long axis of the tooth body. The positive z-axis direction was oriented toward the gingival side, the positive x-axis direction toward the mesial side, and the positive y-axis direction toward the lingual side.

### Finite element simulation and result analysis

The maximum displacement values of the crowns and roots and the average displacement values of the teeth in mandibular dentition were extracted for result analysis. The ratios of the maximum displacement of the crowns and roots were calculated and named as C/R ratios. Furthermore, the average displacement values of premolars and anterior teeth were calculated.

## Results

**FIGURE 4:**
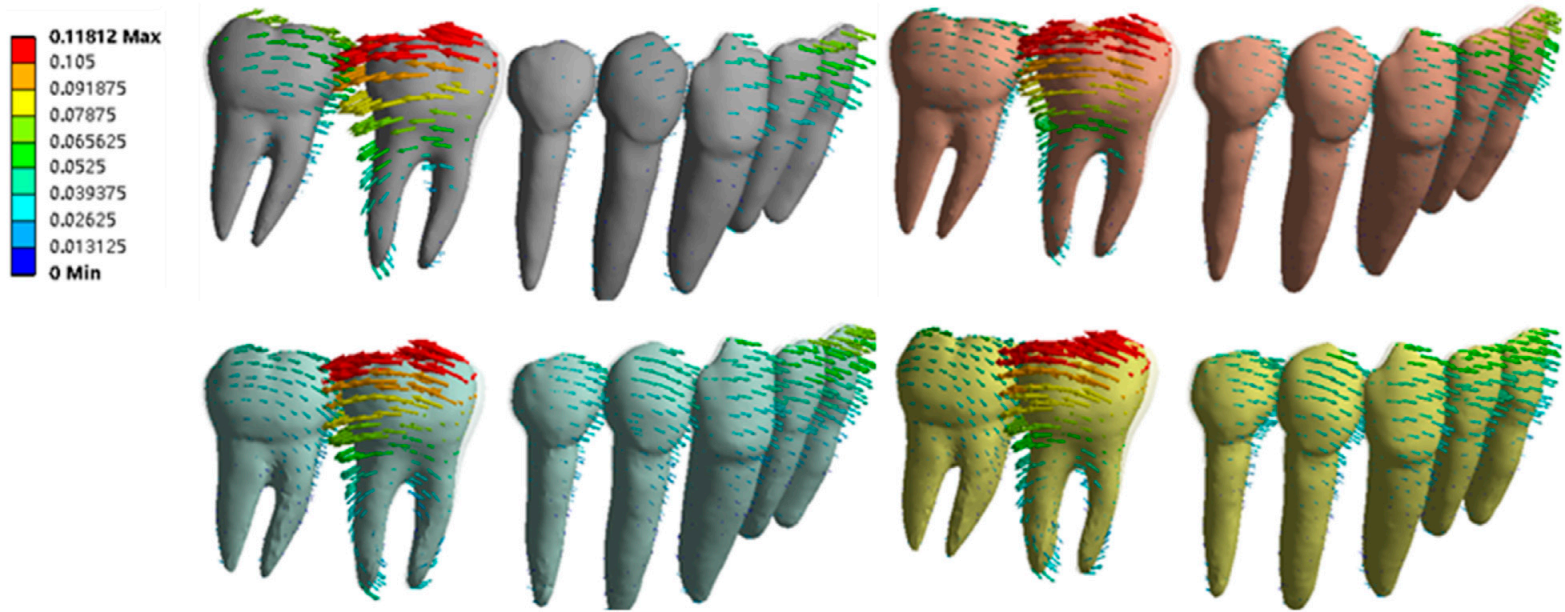
Displacement vector graph of the NO ATT group under four settings.

During the distalization of the mandibular first molar, all teeth exhibited tipping movement, and the values of the displacement were recorded, as depicted in Figure 4. The maximum displacement of the tooth crown was at the cusp or incisal edge, while that of the root was at the root apex. The first molar showed the largest tipping movement in all groups. During the distal tipping movement of the first molar, the second molar and premolar showed mesial tipping movement, and the anterior teeth exhibited labial tipping movement.

**FIGURE 5:**
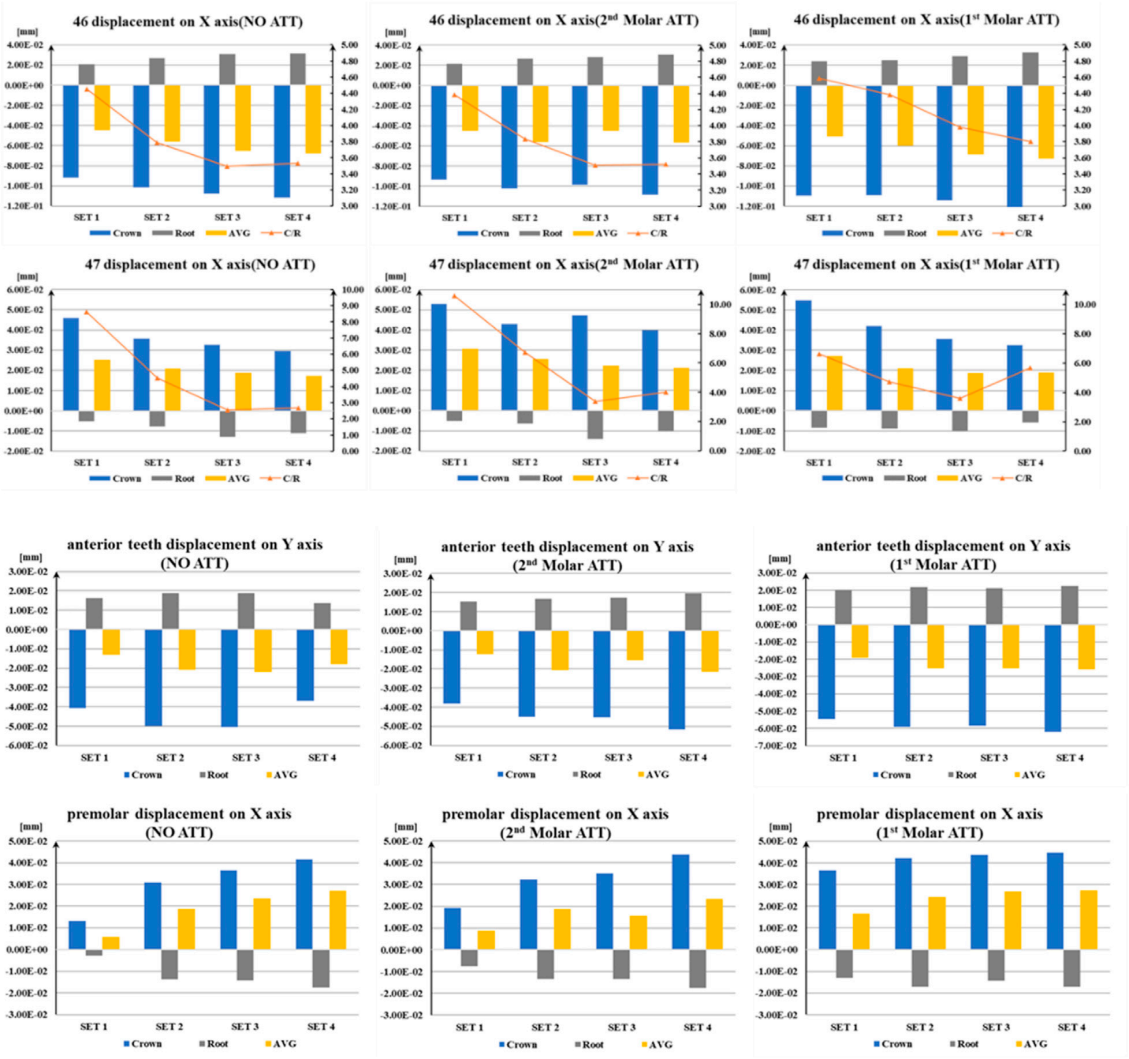
Directional displacement of molars, premolars, and anterior teeth.

The quantitative data of tooth displacement are shown in Figure 5 (Supplementary Tables 1–12). Due to the symmetrical characteristics of both sides of the mandible arches, the right side of the mandible was selected for analysis. With the same attachment configuration, the average, crown, and root displacement of the first molar (46) increased, whereas those of the second molar (47) decreased from SET 1 to SET 4. Regarding different attachment configurations, the average displacement of the first molar in the first molar ATT group was higher than that of the NO ATT group and the second molar ATT group. The maximum average of the first molar ranged from 5.06e^−2^ mm (SET 1) to 7.26e^−2^ mm (SET4). For the minimum average displacement, it was 4.46e^−2^ mm and 5.58e^−2^ mm in SET 1 and SET 2 in the NO ATT group, respectively, and 4.51e^−2^ mm and 5.69e^−2^ mm in SET 3 and SET 4 in the second molar ATT group, respectively. The second molar reached the maximum average displacement of 3.07e^−2^ mm, 2.57e^−2^ mm, 2.23e^−2^ mm, and 2.12e^−2^ mm from SET 1 to SET 4, respectively, in the second molar ATT group. For the minimum average displacement, it was 2.51e^−2^ mm, 2.09e^−2^ mm, 1.88e^−2^ mm, and 1.74e^−2^ mm from SET 1 to SET 4, respectively, in the No ATT group. In the first molar ATT group, the displacement of the second and first molar crowns reached the maximum values at 5.48e^−2^ mm and 1.10e^−1^ mm, respectively, in SET 1, and their ratio was close to 0.5. However, in SET 4, the corresponding values were 3.25e^−2^ mm and 1.24e^−1^ mm, and the ratio decreased to 0.26.

The variation patterns in the C/R ratio were consistent among all groups except for the first molar in the first molar ATT group, decreasing from SET 1 to SET 3 and then increasing from SET 3 to SET 4. The ratio of C/R achieved minimum values in SET 3, with the corresponding values at 3.5 for the first molar and 2.5 for the second molar in the NO ATT group and 3.5 for the first molar and 3.3 for the second molar in the second molar ATT group. In the first molar ATT group, the C/R ratio of the first molar decreased from SET 1 to SET 4 and reached the minimum value at 3.8 in SET 4. The ratio of C/R achieved the maximum values in SET 1 in all groups, with values at 4.4, 4.4, and 4.6 for the first molar and 8.6, 10.1, and 6.6 for the second molar in the NO ATT group, second molar group, and first molar ATT group, respectively.

The mesial displacement of premolars and labial displacement of anterior teeth increased from SET 1 to SET 4, except for the anterior teeth in the NO ATT group, reaching the minimum value in SET 4, as shown in Figure 5. The average displacement of anterior teeth and premolars in the first molar ATT group was more than that of the other groups. Similar to the displacement variation patterns of the first molar shown in Figure 5, which increased from SET 1 to SET 4, the displacement of the anterior tooth crown (6.19e^−2^ mm) and premolar crown (4.48e^−2^ mm) reached the maximum value in SET 4 of the first molar ATT group. The labial displacement of anterior teeth was more than the mesial displacement of premolars, with the corresponding maximum values at 5.18e^−2^ mm of anterior teeth and 4.37e^−2^ mm of premolars in SET 4 of the second molar ATT group.

## Discussion

The study found that the distal displacement of the first molar increased progressively during its distal movement from the initial position. Moreover, the maximal distal displacement of the first molar was achieved by placing the horizontal rectangular attachment on itself.

Finite element analysis (FEA) is an effective numerical technique used to simulate tooth movement (Vatansever et al., 2025). It was first applied in orthodontics by Takahashi et al. (1980) and has since become one of the main methods for biomechanical analysis. Although the acquisition of reliable results is predetermined by the accurate modeling and precise material properties (Fill et al., 2012; Cattaneo and Cornelis, 2021; Ding et al., 2015), a proper balance between the accuracy and simplifications of the material properties of the PDLs should be introduced without compromising the results to quickly estimate orthodontic tooth movement during treatment planning (Ahmed et al., 2023; Tang et al., 2025; Cerne and Petkovsek, 2022). In this study, a 3D finite element model of mandibular first molar distalization by CAs was constructed with the following characteristics (Park et al., 2024). It was the first study examining the distal displacement of the mandibular first molar under varying initial positions and attachment configurations (Inchingolo et al., 2023). A second-order tetrahedral element mesh was designed, exhibiting no significant distortion in the analysis outcomes (Martina et al., 2024). The stress extracted from CAs when the teeth were returned from the target positions to the initial positions was applied to the pre-orthodontic dentition and served as the orthodontic force, approximating the actual force application of the CAs.

Because the mandibular first molar is the occlusion key in mandible dentition, achieving a neutral molar occlusal relationship is essential (Murugan et al., 2024). For cases that adjust the occlusal relationship through mandibular molar distalization, the course of treatment mainly depends on the efficiency of mandibular first molar distalization, which is the lowest among all teeth in both arches (Wu et al., 2021). Therefore, to improve the overall treatment efficiency, we investigated the distalization of the mandibular first molar at varying initial positions at the same step distance (0.2 mm). The results showed that the distal movement of the first molar’s initial position increased the first molar displacement. On one hand, it can be explained by the co-action of compressed CA material mesial to the first molar and the expanded CA material distal to the first molar. When the CA was positioned on the dental arch, the deformation of the CAs mesial and distal to the first molar was the same. Hooke’s Law stated that within the elastic limit, the stress applied to a material was directly proportional to the strain it produced (Noh et al., 2024). With the same deformation (change), the original length of CAs is negatively co-related to the strain (Wang et al., 2021). Therefore, the largest stress appeared in SET 4, when the original length of CA was the shortest and the strain was the highest. From SET 1 to SET 4, the CA material distal to the first molar became shorter and played an increasingly significant role, thus leading to an increased efficiency of mandibular first molar distalization. On the other hand, this finding would make sense in orthodontic clinical practice because the limited space in the interdental gap in SET 1 weakens the compression from the mesial CA materials. Moreover, the increased contact area between the CAs and the teeth from SET 1 to SET 4 contributed to the efficiency of molar distalization. Based on these findings, an increased step distance in precedence can be designed to enhance the overall movement efficiency.

In CA treatment, attachments assist retention, enhance the application of orthodontic force, and promote the precision of tooth movement by increasing the contact area between the CAs and the teeth (AlMogbel, 2023; Putr et al., 2021). They are strategically placed on the tooth according to specific tooth movements, such as rotation, extrusion, and bodily movement. In this study, the highest efficiency of first molar horizontal distalization was achieved when the rectangular attachment was positioned on the first molar because the better grip, increased frictional force, and the couple of forces were applied to it (Ren et al., 2022). Consequently, placing a horizontal rectangular attachment on the first molar may shorten treatment time or improve predictability. The above-mentioned finding can serve as a strategy for attachment configurations in treatment design.

Considerations regarding anchorage control are revealed in this study. The stages of molar distalization showed a V-pattern, which can be either the sparse type or the intensive type. The sparse type, in which CAs distalize the molars, premolars, and incisors one by one, was selected in this study as it may theoretically cause less opposite movement of other teeth than the intensive type, in which CAs distalize several teeth simultaneously. However, the results showed that the opposite movement of other teeth was observed during molar distalization. This indicates that the application of additional anchorage, such as a mini-implant or inter-dentition Class III elastics (He et al., 2013), is required to prevent adverse effects in certain cases.

*In vivo* and *in vitro* experiments are required to investigate the effects of biological remodeling. Further validation of the results of this study necessitates exploration in the treatment design of clinical practice.

## Conclusion

Without additional anchorage, the distal displacement of the first molar by CAs is concomitant with the opposite movement of other teeth in the dentition.

The maximum distal displacement of the first molar crown can be achieved by placing a horizontal rectangular attachment on itself.

With the distal movement of the first molar’s initial position, the movement efficiency of the mandibular first molar was increased. Therefore, increased step distance in precedence can be designed to enhance the overall movement efficiency.

## Limitations

Although finite element analysis offers numerous benefits, it is essential to be aware of its limitations to ensure proper application. First, all the results were based on simplified assumptions; for instance, the simplified PDL properties may not fully capture the actual characteristics of the system. Moreover, the lack of biological remodeling may affect the reliability of the results.

## Data Availability

The original contributions presented in the study are included in the article/supplementary material; further inquiries can be directed to the corresponding authors.
