# Correction: Distalization of the mandibular first molar with clear aligners: a 3D finite element study

**DOI:** 10.3389/fbioe.2025.1747668

**Published:** 2025-11-28

**Authors:** Fujia Kang, Yifei Xu, Xinyan Zhao, Zhen Lu, Dongning Li, Jiamin Zhao, Yibao Li, Menghong Li, Kun Qi

**Affiliations:** 1 Key Laboratory of Shaanxi Province for Craniofacial Precision Medicine Research, College of Stomatology, Xi’an Jiaotong University, Xi’an, Shaanxi, China; 2 Department of Orthodontics, College of Stomatology, Xi’an Jiaotong University, Xi’an, Shaanxi, China; 3 Department of Oral Anatomy and Physiology and TMD, School of Stomatology, Air Force Medical University, Xi’an, Shaanxi, China; 4 School of Mathematics and Statistics, Xi’an Jiaotong University, Xi’an, Shaanxi, China

**Keywords:** clear aligner, tooth movement, molar distalization, finite element analysis, orthodontics

Affiliations 1 and 2 were erroneously given as “1Hospital of Stomatology, Xi’an Jiaotong University, Xi’an, Shaanxi, China,

2Department of Stomatology, Xi’an Jiaotong University, Xi’an, Shaanxi, China.”

Affiliations 1 and 2 have been corrected to “1Key laboratory of Shaanxi Province for Craniofacial Precision Medicine Research, College of Stomatology, Xi’an Jiaotong University, Xi’an, Shaanxi, China.

2Department of Orthodontics, College of Stomatology, Xi’an Jiaotong University, Xi’an, Shaanxi, China”.

The original article has been updated.

