# Supplementary material for: Distalization of the mandibular first molar with clear aligners: a 3D finite element study
Source: Front Bioeng Biotechnol. 2025 Nov 13;13:1665588. doi: 10.3389/fbioe.2025.1665588 (PMC12657459; doi:10.3389/fbioe.2025.1665588)
Supplement: Supplementary file 1 [file DataSheet1.docx]

Tab.1 46 displacement on X axis (NO ATT)

|  | Crown | Root | AVG | C/R |
| --- | --- | --- | --- | --- |
| SET 1 | -9.18E-02 | 2.06E-02 | -4.46E-02 | 4.45 |
| SET 2 | -1.01E-01 | 2.68E-02 | -5.58E-02 | 3.79 |
| SET 3 | -1.08E-01 | 3.08E-02 | -6.53E-02 | 3.49 |
| SET 4 | -1.12E-01 | 3.16E-02 | -6.79E-02 | 3.54 |

Tab.2 46 displacement on X axis (2^nd^ Molar ATT)

|  | Crown | Root | AVG | C/R |
| --- | --- | --- | --- | --- |
| SET 1 | -9.34E-02 | 2.13E-02 | -4.51E-02 | 4.38 |
| SET 2 | -1.02E-01 | 2.67E-02 | -5.61E-02 | 3.83 |
| SET 3 | -9.87E-02 | 2.81E-02 | -4.51E-02 | 3.51 |
| SET 4 | -1.09E-01 | 3.08E-02 | -5.69E-02 | 3.52 |

Tab.3 46 displacement on X axis (1^st^ Molar ATT)

|  | Crown | Root | AVG | C/R |
| --- | --- | --- | --- | --- |
| SET 1 | -1.10E-01 | 2.39E-02 | -5.06E-02 | 4.59 |
| SET 2 | -1.09E-01 | 2.49E-02 | -5.98E-02 | 4.39 |
| SET 3 | -1.14E-01 | 2.87E-02 | -6.84E-02 | 3.98 |
| SET 4 | -1.24E-01 | 3.27E-02 | -7.26E-02 | 3.80 |

Tab.4 47 displacement on X axis (NO ATT)

|  | Crown | Root | AVG | C/R |
| --- | --- | --- | --- | --- |
| SET 1 | 4.59E-02 | -5.31E-03 | 2.51E-02 | 8.63 |
| SET 2 | 3.58E-02 | -7.89E-03 | 2.09E-02 | 4.54 |
| SET 3 | 3.25E-02 | -1.28E-02 | 1.88E-02 | 2.55 |
| SET 4 | 2.97E-02 | -1.10E-02 | 1.74E-02 | 2.69 |

Tab.5 displacement on X axis (2^nd^ Molar ATT)

|  | Crown | Root | AVG | C/R |
| --- | --- | --- | --- | --- |
| SET 1 | 5.30E-02 | -5.01E-03 | 3.07E-02 | 10.57 |
| SET 2 | 4.30E-02 | -6.40E-03 | 2.57E-02 | 6.72 |
| SET 3 | 4.73E-02 | -1.40E-02 | 2.23E-02 | 3.37 |
| SET 4 | 3.98E-02 | -9.94E-03 | 2.12E-02 | 4.00 |

Tab.6 47 displacement on X axis (1^st^ NO ATT)

|  | Crown | Root | AVG | C/R |
| --- | --- | --- | --- | --- |
| SET 1 | 5.48E-02 | -8.29E-03 | 2.70E-02 | 6.61 |
| SET 2 | 4.19E-02 | -8.89E-03 | 2.11E-02 | 4.72 |
| SET 3 | 3.57E-02 | -9.88E-03 | 1.87E-02 | 3.61 |
| SET 4 | 3.25E-02 | -5.72E-03 | 1.89E-02 | 5.69 |

Tab.7 anterior teeth displacement on Y axis (NO ATT)

|  | Crown | Root | AVG |
| --- | --- | --- | --- |
| SET 1 | -4.07E-02 | 1.62E-02 | -1.32E-02 |
| SET 2 | -4.48E-02 | 1.89E-02 | -2.07E-02 |
| SET 3 | -4.55E-02 | 1.89E-02 | -2.19E-02 |
| SET 4 | -4.69E-02 | 1.37E-02 | -1.80E-02 |

Tab.8 anterior teeth displacement on Y axis (2^nd^ Molar ATT)

|  | Crown | Root | AVG |
| --- | --- | --- | --- |
| SET 1 | -3.82E-02 | 1.52E-02 | -1.24E-02 |
| SET 2 | -4.50E-02 | 1.67E-02 | -2.05E-02 |
| SET 3 | -4.52E-02 | 1.74E-02 | -1.53E-02 |
| SET 4 | -5.18E-02 | 1.95E-02 | -2.14E-02 |

Tab.9 anterior teeth displacement on Y axis (1^st^ NO ATT)

|  | Crown | Root | AVG |
| --- | --- | --- | --- |
| SET 1 | -5.47E-02 | 2.02E-02 | -1.92E-02 |
| SET 2 | -5.92E-02 | 2.18E-02 | -2.52E-02 |
| SET 3 | -5.83E-02 | 2.10E-02 | -2.52E-02 |
| SET 4 | -6.19E-02 | 2.25E-02 | -2.59E-02 |

Tab.10 premolar displacement on X axis (NO ATT)

|  | Crown | Root | AVG |
| --- | --- | --- | --- |
| SET 1 | 1.32E-02 | -2.84E-03 | 5.83E-03 |
| SET 2 | 3.08E-02 | -1.38E-02 | 1.87E-02 |
| SET 3 | 3.66E-02 | -1.42E-02 | 2.36E-02 |
| SET 4 | 4.16E-02 | -1.76E-02 | 2.70E-02 |

Tab.11 premolar displacement on X axis (2^nd^ Molar ATT)

|  | Crown | Root | AVG |
| --- | --- | --- | --- |
| SET 1 | 1.93E-02 | -7.49E-03 | 8.87E-03 |
| SET 2 | 3.23E-02 | -1.35E-02 | 1.88E-02 |
| SET 3 | 3.50E-02 | -1.35E-02 | 1.57E-02 |
| SET 4 | 4.37E-02 | -1.75E-02 | 2.34E-02 |

Tab.12 premolar displacement on X axis (1^st^ NO ATT)

|  | Crown | Root | AVG |
| --- | --- | --- | --- |
| SET 1 | 3.64E-02 | -1.31E-02 | 1.67E-02 |
| SET 2 | 4.21E-02 | -1.72E-02 | 2.43E-02 |
| SET 3 | 4.36E-02 | -1.44E-02 | 2.69E-02 |
| SET 4 | 4.48E-02 | -1.71E-02 | 2.72E-02 |
